# Wastewater-based epidemiology reveals spatiotemporal dynamics and diurnal temperature range effects on post-pandemic SARS-CoV-2 infection burden in Chongqing, China

**DOI:** 10.3389/fpubh.2026.1891965

**Published:** 2026-08-26

**Authors:** Yi Li, Zihao Wang, Ju Wang, Qi Zhang, Jiqin Zheng, Yu Xiong, Hui Zeng, Sheng Ye, Deqiang Mao, Huadong Zhang

**Affiliations:** Chongqing Municipal Center for Disease Control and Prevention, Chongqing Institute of Preventive Medicine, Chongqing, China

**Keywords:** diurnal temperature range, N gene viral load, post-pandemic, SARS-CoV-2, spatiotemporal heterogeneity, wastewater-based epidemiology

## Abstract

**Background:**

Wastewater-based epidemiology (WBE) is a cornerstone of post-pandemic SARS-CoV-2 surveillance, yet the environmental covariates and spatiotemporal heterogeneities of viral signals remain poorly characterized.

**Methods:**

We conducted a 35-month longitudinal study across 19 sites in Chongqing, China, quantifying SARS-CoV-2N gene concentrations via RT-qPCR to assess the value of WBE and the correlation between viral concentrations and meteorological fluctuations. We employed Gaussian generalized additive models with REML estimation to quantify seasonal dynamics and lagged effects of diurnal temperature range (DTR).

**Results:**

A total of 2,520 wastewater samples with complete data were obtained, with an overall N gene detection rate of 62.02%, a median flow-population normalized viral load of 8.40 log10 gc/day per 1,000 inhabitants, and median 1-day, 3-day and 7-day lagged DTR of 6.05 °C, 6.05 °C and 5.67 °C, respectively. Our results demonstrate a surveillance lead time for wastewater signals relative to clinical notifications. WBE detected a 2025 resurgence 2 months earlier than official reports. Wastewater SARS-CoV-2 circulation exhibited a distinct seasonal pattern, characterized by a primary peak in June and two secondary peaks in March and August. The spatiotemporal distribution patterns revealed by monthly heatmaps were highly consistent with the city-wide seasonal trends, and significant spatial heterogeneity was observed. The 7-day moving average DTR exhibited a complex nonlinear relationship with viral loads, initially decreasing, then increasing to a peak at 7–8 °C before declining again.

**Conclusion:**

These findings indicate that WBE serves as a useful population-level proxy for infection burden and trends, complementing clinical surveillance. The statistical association between DTR and viral dynamics offers a predictive framework for refining early-warning systems and informs targeted interventions in complex urban environments.

## 1 Introduction

The World Health Organization officially terminated the COVID-19 Public Health Emergency of International Concern in May 2023 (1). Since then, SARS-CoV-2 has become a persistent public health challenge managed under routine surveillance and control measures. China subsequently reclassified COVID-19 as a Category B infectious disease. To date, SARS-CoV-2 transmission continues to evolve and fluctuate (2, 3). Traditional case surveillance has become incomplete when it comes to detecting changes in testing willingness and healthcare-seeking behavior (3–6).

The COVID-19 pandemic prompted over 70 countries to establish large-scale wastewater surveillance networks (7), marking substantial progress in the field (8–11). Early applications were largely confined to the emergency phase of the pandemic and faced considerable uncertainties in estimating SARS-CoV-2 prevalence (12–14). These uncertainties include whether the viral load in sewage can serve as an indicator of actual infection, as well as interference from factors such as the matrix and flow rate in the sewage. Subsequent evidence has demonstrated that wastewater data can indeed reflect genuine community infection dynamics and enable the earlier detection of COVID-19 variants (15–19). WBE has since been widely regarded as a key tool for complementing clinical case reporting (20–22). Most evidence has come from the pandemic response phase, and the role of WBE in post-pandemic surveillance remains insufficiently quantified relative to clinical systems. Moreover, wastewater research has focused primarily on temporal variation, with limited attention to the meteorological covariates and spatial heterogeneity that may help predict observed fluctuations in wastewater viral loads (2).

The seasonality of COVID-19 transmission remains debated. Although temperature and humidity have been found to be only modestly associated with SARS-CoV-2 infection burden (23–27), long-term wastewater genomic surveillance over the same period has consistently revealed seasonal variation in community circulation, with peaks arising in different seasons and frequently driven by variant-specific dominance (2, 28, 29). This apparent discrepancy highlights the need to consider additional meteorological dimensions beyond mean temperature and humidity. Modeling studies based on a physicochemical perspective have suggested that DTR may predict SARS-CoV-2 lifetimes (30). Consistently, epidemiological studies have reported associations between DTR and COVID-19 transmission risk, including evidence from a global panel dataset spanning 58 cities and independent observations from China (31, 32). Nevertheless, most epidemiological evidence relies on notifications of clinical cases, which decreased in the post-pandemic period, and the relationship between DTR and SARS-CoV-2 viral loads in wastewater has not yet been systematically evaluated.

In this study, using long-term wastewater surveillance data from Chongqing, we characterized the temporal dynamics and seasonal structure of SARS-CoV-2 concentrations and assessed the incremental value of WBE for post-pandemic COVID-19 monitoring beyond clinical indicators. We further examined spatiotemporal heterogeneity in local transmission and quantified the nonlinear lagged effects of DTR on wastewater viral signals.

## 2 Materials and methods

### 2.1 Study design

This study established a wastewater-based epidemiology surveillance network across 16 districts of Chongqing Municipality, which was rolled out and expanded in phases from February 2023 to January 2025. The network was initially launched with 5 core municipal wastewater treatment plants (WWTPs) in the main urban area, selected as the top 5 facilities by treatment capacity and service population. Following the overall planning of the city-wide public health surveillance program, the network was expanded stepwise alongside improvements in laboratory testing capacity. Ultimately, the surveillance network covered 18 urban WWTPs and 1 sewage pumping station, with a combined daily treatment capacity of 2.3 million tonnes and a service population of 11 million (detailed phased expansion information is provided in Supplementary Table S1). This sewage pumping station without wastewater treatment facilities. It was treated as an equivalent WWTP site and included in all analyses, as it serves as the sole wastewater collection hub for the corresponding district. Wastewater samples were collected at facility inlets to quantify SARS-CoV-2 in raw sewage as a proxy for community infection. We only documented basic information for each site, including geographic location and service population. The served population of the WWTPs ranged from 150,000 to 3,000,000 persons, with six WWTPs serving populations exceeding 500,000 persons. Prior to November 2024, 24 h composite samples were collected twice weekly (Mondays and Wednesdays) via automatic samplers and delivered to regional central laboratories for analysis on Tuesdays and Thursdays, respectively. Afterward, sampling was reduced to once weekly (Mondays only), with delivery and analysis on Tuesdays.

Wastewater samples were collected as 24-h composite influent samples using refrigerated automatic samplers, with each sampling cycle running continuously from 8:00 a.m. to 8:00 a.m. the next day. 200 mL of wastewater was collected every hour, yielding a total composite volume of 4,800 mL over 24 h. After completion of sampling, the composite sample was thoroughly mixed, and a 1,000 mL aliquot was transferred into sterile sampling bottles and transported to the laboratory for subsequent SARS-CoV-2 analysis. All samples were kept at 0 °C ~ 4 °C under cold chain conditions during collection, transportation, and storage before laboratory processing. Samples were delivered to the laboratory within 2 h and processed for analysis within 24 h.

Wastewater physicochemical parameters during the sampling period, including daily treatment flow, influent water temperature, total suspended solids, chemical oxygen demand, pH, and ammonia nitrogen, were obtained from the online monitoring systems of the corresponding wastewater treatment plants. Continuous monitoring data were recorded over the same 24-h sampling period, and the values used in the analysis were calculated as 24-h averages. This study collected monthly statistics on reported incidence of legally notifiable infectious diseases in Chongqing Municipality from November 2023 to December 2025, sourced from the Chongqing Disease Surveillance Network. Daily meteorological data were sourced from the Chongqing Meteorological Bureau. The daily weather data is sourced from the Chongqing Meteorological Bureau’s weather stations.

### 2.2 Detection methods for SARS-CoV-2 in sewage

All viral enrichment, nucleic acid extraction and real-time fluorescent reverse transcription polymerase chain reaction (RT-qPCR) quantification procedures complied with two national surveillance standards WS/T 799–2022 (Method for enrichment and nucleic acid detection of SARS-CoV-2 in sewage) and WS/T 10042–2025 (Technical specification for surveillance of SARS-CoV-2 in wastewater). Due to variable instruments and reagent stocks among Chongqing’s district laboratories, each site adopted tailored testing workflows including four optional viral enrichment protocols (aluminum flocculation precipitation, polyethylene glycol (PEG) precipitation, centrifugal ultrafiltration, and magnetic bead-based enrichment.), matched RNA extraction systems, and different RT-qPCR platforms and SARS-CoV-2 detection kits. The complete instrument and reagent information is listed in Supplementary Table S4. The lower limits of detection (LOD) are 10^0^ copies/mL for the PEG, aluminum salt and ultrafiltration method, and 10^1^ copies/mL for the magnetic bead method.

Dual-target RT-qPCR targeting ORF1ab and N genes was performed. Extracted nucleic acid samples were subjected to amplification system preparation and RT-qPCR detection following the manufacturer’s instructions of the RT-qPCR kit. Considering the superior stability and longer persistence of the N gene in sewage matrices (33), N gene-derived viral loads were used to represent community-wide SARS-CoV-2 burden. PMMoV was quantified as the mandatory internal standard specified in WS/T 10042–2025 to calculate sample-specific recovery rates for viral load correction. Full step-by-step laboratory protocols are documented in Supplementary Text S1.

Between February 2023 and December 2025, a total of 2,547 wastewater samples were collected. After excluding samples with missing N gene detection results (*n* = 2) or daily wastewater flow data (*n* = 25), 2,520 samples with complete data were included in the final analysis. The number of samples for PEG precipitation, centrifugal ultrafiltration, aluminum flocculation precipitation and magnetic bead method was 1,057, 202, 1,100 and 161, respectively.

### 2.3 Variable definition

The wastewater indicator used in this study was the population-normalized viral load, defined as the number of viral genome copies discharged per 1,000 inhabitants per day. This indicator normalizes for differences in wastewater flow rates and served population sizes across WWTPs, enabling valid spatiotemporal comparisons. All non-detects were imputed as one-half of the limit of detection (1/2 LOD).

#### 2.3.1 Flow- and population-weighted viral load normalization

Raw SARS-CoV-2 concentrations were further normalized by wastewater flow and service population to obtain weighted viral loads. For each WWTP sample, the standardized viral load, V, was calculated as follows (Equation 1):

$$V=\frac{C\times Q\times 1000}{P}$$ (1)

Where C is the concentration of SARS-CoV-2 N gene in the wastewater sample (gc/L), Q is the daily wastewater influent flow rate at the time of sampling (L/day), and P is the population served by the corresponding WWTP (inhabitants). The factor 1,000 normalizes the result to per 1,000 inhabitants.

#### 2.3.2 Monthly district-level weighted viral load

To obtain a representative monthly viral load for each district, we first calculated the average viral flux for each WWTP across all samples collected in that month. The district-level viral load was then computed by summing the average fluxes from all WWTPs in the district and normalizing to the total served population (Equation 2):

$$V_{i,m}=\frac{\sum_{j=1}^{n_{i}}(\frac{1}{k_{j,m}}\sum_{t=1}^{k_{j,m}}C_{i,j,t}\times Q_{i,j,t})\times 1000}{\sum_{j=1}^{n_{i}}P_{i,j}}$$ (2)

$V_{i,m}$ is the weighted viral load for the i-th district in the m-th month.

$n_{i}$ is the number of WWTPs in the i-th district.

$k_{j,m}$ is the number of samples collected from the j-th WWTP in the m-th month.

All viral load values were log_10_(x) transformed to improve normality for subsequent statistical analyses. Seasonal trends were summarized using monthly weighted averages to account for minor differences in sampling frequency across the study period.

#### 2.3.3 Estimation of the infected population

By correlating measured wastewater viral loads with the average daily viral shedding rate per infected individual, the infected population can be quantified (Equation 3). Considering that pepper mild mottle virus (PMMoV) monitoring was initiated in March 2025, infected population estimation was restricted to the period thereafter. To ensure a conservative estimation and avoid overstating the actual infection burden, all fixed parameters were set to higher values from established wastewater surveillance literature.

The formula for estimating the infected population is as follows (34–36):

$$N_{\text{infected}}=\frac{Q\times C}{p\times V_{\text{feces}}\times M_{\text{feces}}\times \eta_{\text{PMMoV}}}$$ (3)

Where:

$N_{\text{infected}}$ is estimated number of infected individuals.

Q is daily wastewater flow rate.

C is wastewater viral concentration.

p is viral shedding probability (82.5%) (37).

$V_{\text{feces}}$ is fecal viral load per gram (450,000,000 (copies/g)) (38).

$M_{\text{feces}}$ is daily fecal mass per person (128 g) (39).

$\eta_{\text{PMMoV}}$ is the laboratory recovery efficiency of PMMoV, used as the viral recovery metric for this estimation.

#### 2.3.4 Meteorological factors and other covariates

This study focused primarily on DTR, calculated as the difference between daily maximum and minimum air temperatures provided by the Chongqing Meteorological Bureau. To evaluate potential lag effects, we defined DTR1lag as the DTR recorded the day prior to wastewater sample collection. We also assessed cumulative lag effects using the average DTR over the 3-day (DTR3lag) and 7-day (DTR7lag) periods preceding each sampling date. Additionally, several physicochemical indicators and operational variables were included as covariates: daily wastewater treatment volume (DWTV), influent wastewater temperature (IWT), chemical oxygen demand (CODCr), pH, suspended solids (SS), ammonia nitrogen (NH_3_-N), and daily service population.

### 2.4 Statistical data analysis

Concentration and normalized weighted load values, together with physicochemical wastewater parameters and their distribution and dispersion characteristics, were summarized for each individual WWTP. DTR across each study administrative district, as well as city-wide officially notified case counts, were also subjected to descriptive statistical analysis, including calculation of minimum, maximum, median, mean and standard deviation.

In descriptive analysis and spatio-temporal distribution visualization, flow-population weighted wastewater viral load was adopted to eliminate heterogeneity in wastewater discharge and service population across different wastewater treatment plants, thereby objectively reflecting the overall community viral shedding level. In this study, we calculated monthly district-level weighted viral load for each calendar month and spatial unit.

Generalized additive models (GAMs) with Gaussian distributions and restricted maximum likelihood (REML) estimation were used to assess seasonal dynamics and the lagged impacts of DTR on wastewater SARS-CoV-2 concentrations (Equation 4). Viral load served as the dependent variable, with DTR1lag, DTR3lag, and DTR7lag as key independent variables. The model incorporated cyclic splines for seasonal trends, categorical controls for year and concentration method, linear adjustments for wastewater physicochemical characteristics and service population, and random intercepts to address district-level clustering. All continuous predictors were Z-score standardized to eliminate scale differences, including meteorological, physicochemical, and demographic variables.

$$\begin{array}{l} \log_{10}N=\alpha +s({\mathrm{DTR}}_{\mathrm{lag}})+s(\text{month},\mathrm{cc})+\text{year}\\ +X\beta +\text{Method}+s(\text{District},\mathrm{re})+\varepsilon \end{array}$$ (4)

Where:

$\log_{10}N$ is log₁₀-transformed raw SARS-CoV-2 N-gene concentration (copies/mL).

α is Model intercept.

$s({\mathrm{DTR}}_{\mathrm{lag}})$ is smoothing function for diurnal temperature range with lagged averaging. DTR_1_: 1-day lag; DTR_3_: 3-day moving average; DTR_7_: 7-day moving average.

s(month,cc) is cyclic cubic regression spline for seasonal variation.

year is categorical variable for inter-annual variation.

Xβ is linear combination of wastewater physicochemical covariates and service population. X includes: DWTV, IWT, SS, CODCr, pH value, NH_3_-N, and service population.

Method is categorical variable for virus concentration method.

s(District,re) is random effect spline for administrative district.

ε is model residual error.

All statistical analyses were conducted in R (version 4.4.2, R Foundation for Statistical Computing, Vienna, Austria). GAMs with REML estimation were fitted via the gam() function from the mgcv package. We adopted cyclic cubic splines (bs = “cc”) for the monthly seasonal term and random intercept splines (bs = “re”) for administrative districts to account for spatial clustering, consistent with Equation 4. Spatial and temporal visualization outputs were generated using ggplot2 and sf packages. Two-sided *p*-values less than 0.05 were considered statistically significant.

## 3 Results

### 3.1 Baseline characteristics

The characteristics of the 19 WWTPs and study samples are summarized in Table 1. The overall detection rate of the N gene was 62.02% (1,563/2520). The flow- and population-normalized viral load ranged from 4.894 to 11.173 log_10_ gc/day per 1,000 inhabitants, with a median of 8.402 log_10_ gc/day per 1,000 inhabitants and an IQR of 1.309 log_10_ gc/day per 1,000 inhabitants.

**Table 1:** Characteristics of wastewater samples, viral concentrations and meteorological variables, February 2023 to December 2025.

Variable	Min	Q1	Median	Q3	Max	IQR	Mean ± SD
log10 normalized N gene concentration (gc/day/1000 inhabitants)	4.894	8.000	8.402	9.309	11.173	1.309	8.64 ± 0.85
Inhabitants served (10^4^ persons)	15.000	31.100	49.000	60.000	300.000	28.900	70.79 ± 76.69
DWTV (10^4^ tons/day)	0.804	5.510	7.521	12.000	123.800	6.490	19.91 ± 29.66
CODCr (mg/L)	17.000	167.000	211.500	279.000	1552.000	112.000	229.38 ± 100.78
pH	4.630	7.460	7.600	7.700	8.300	0.240	7.57 ± 0.24
SS (mg/L)	13.000	104.000	144.000	196.000	2814.790	92.000	214.04 ± 320.53
NH_3_-N (mg/L)	0.220	20.100	24.600	29.600	96.210	9.500	25.17 ± 8.12
IWT (°C)	5.162	15.200	18.700	23.800	32.200	8.600	19.19 ± 5.45
1-day lag DTR	1.100	3.850	6.050	8.300	13.700	4.450	6.18 ± 2.74
3-day lag MA DTR	1.417	4.567	6.050	7.267	13.900	2.700	6.1 ± 2.19
7-day lag MA DTR	1.807	4.821	5.671	7.036	12.743	2.214	6.08 ± 1.93

**Figure 1:**
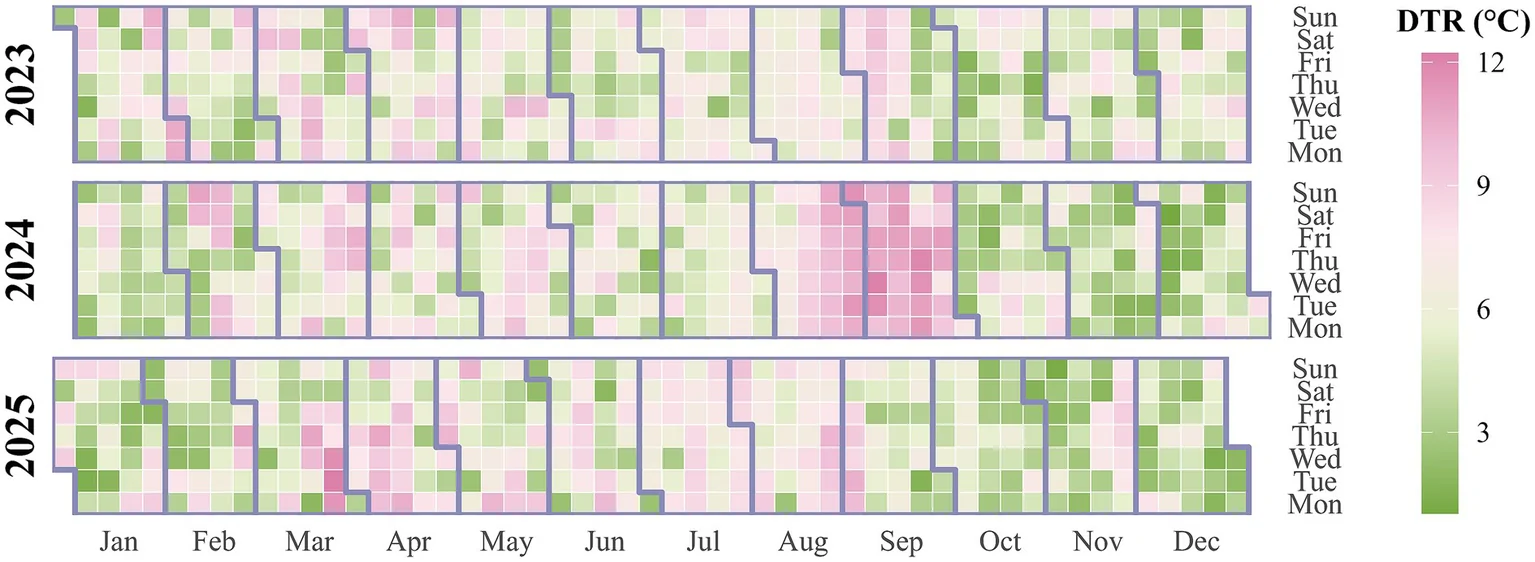
Calendar heatmap of diurnal temperature range in Chongqing (2023–2025).

For meteorological variables, the 1-day lagged DTR ranged from 1.100 to 13.700 °C, with a median of 6.050 °C (IQR: 4.450 °C). The 3-day lagged moving average (MA) DTR varied from 1.417 to 13.900 °C, with a median of 6.050 °C (IQR: 2.700 °C). The 7-day lagged MA DTR ranged from 1.807 to 12.743 °C, with a median of 5.671 °C (IQR: 2.214 °C). The characteristics of each WWTP are presented in Supplementary Table S2, S3. The temporal distribution of DTR is illustrated in Figure 1.

### 3.2 Temporal trends and spatial heterogeneity of wastewater viral load

**Figure 2:**
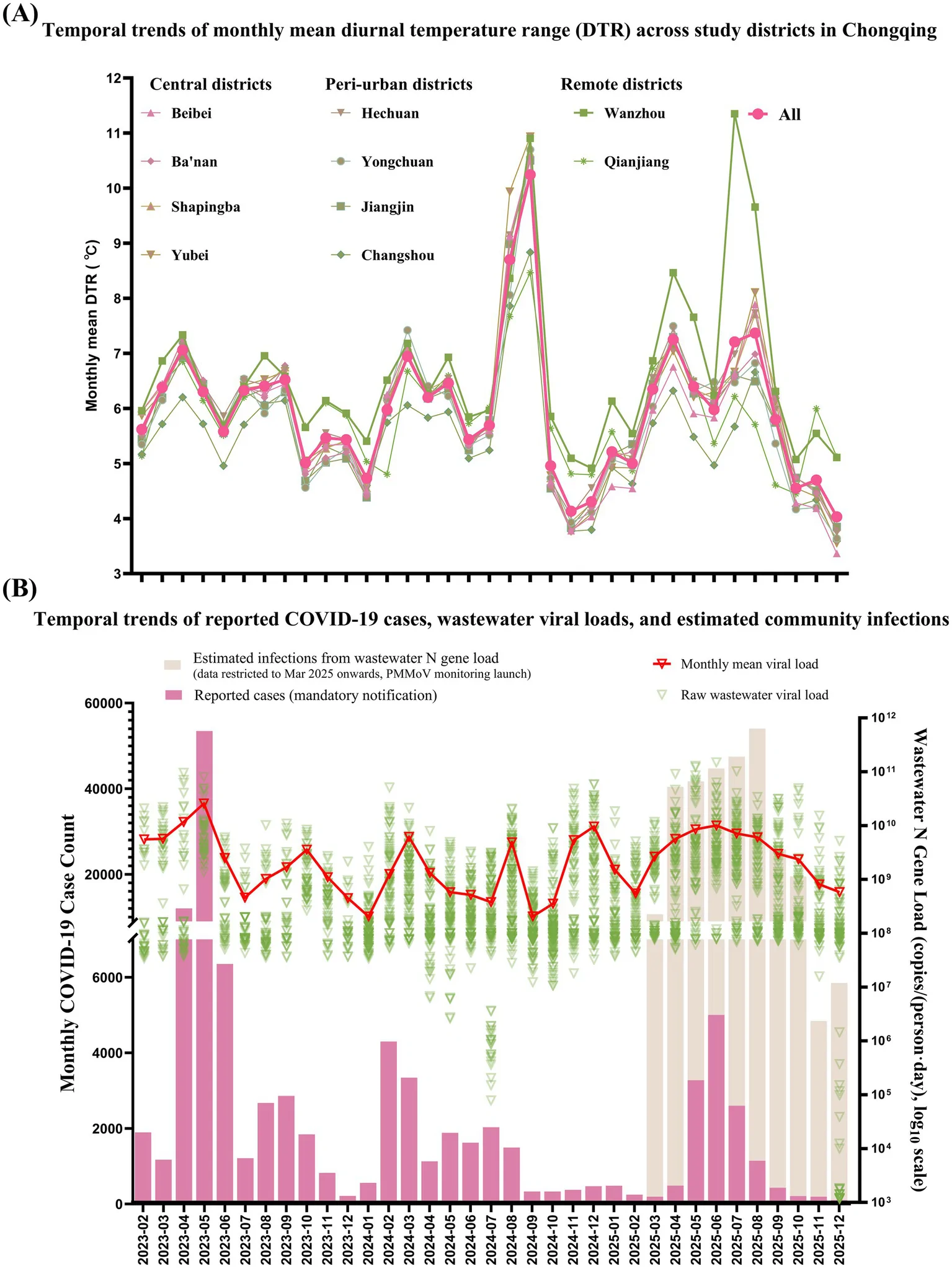
Monthly temporal patterns of DTR, wastewater SARS-CoV-2 viral loads, reported cases, and estimated infections (2023–2025). **(A)** Monthly average DTR stratified by central, peri-urban and remote districts. Separate lines represent individual districts, and the solid rose pink line indicates city-wide mean DTR. **(B)** Dual-axis time series. The left vertical axis presents monthly epidemiological metrics. Dusty rose bars represent officially notified COVID-19 cases, and light beige bars denote estimated infections from normalized wastewater viral loads (data available only from March 2025 following PMMoV internal standard correction). The right log_10_-scaled vertical axis displays weighted SARS-CoV-2 N gene concentrations, with olive green downward triangles indicating raw daily weighted viral loads and red triangles with lines showing monthly district-averaged viral loads.

From 2023 to 2024, the monthly average DTR across all study districts and counties exhibited a highly synchronized seasonal fluctuation pattern. During April–May and July–August 2025, the monthly DTR in the outlying district of Wanzhou remained consistently higher than the city-wide average. From 2023 to the first half of 2024, the trends in monthly cases and viral load in wastewater were highly coincident. From September 2024 to April 2025, officially reported case numbers remained at extremely low levels with virtually no fluctuation. However, viral load in wastewater continued to exhibit clear cyclical fluctuations. From May 2025 onwards, the trends in reported cases and wastewater viral load once again became synchronized (see Figure 2). In fact, viral loads in wastewater had already begun to rise as early as March 2025.

**Figure 3:**
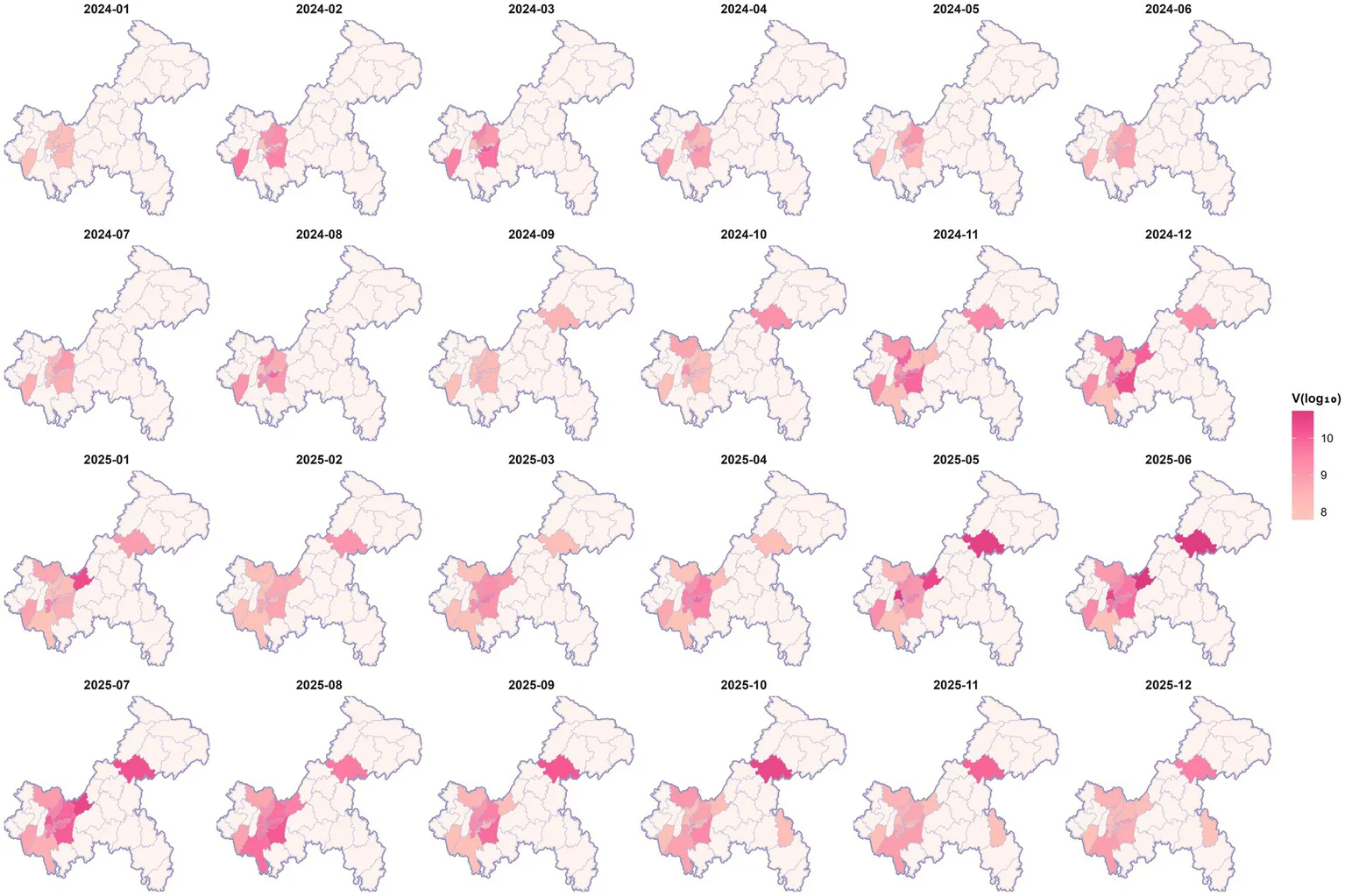
Monthly spatio-temporal distribution of wastewater SARS-CoV-2 viral load across districts in Chongqing, 2024–2025. *V(log_10_) is the monthly district-level weighted viral load.

Monthly spatio-temporal heatmaps revealed distinct patterns of community viral circulation from 2024 to 2025 (Figure 3). In 2024, there was an observation of high peaks of SARS-CoV-2 N gene viral load mainly in central urban areas. The first outbreak peak occurred in February–March, whereas the second smaller outbreak occurred in August, both of which were geographically restricted to the central urban areas. A marked trend of spatial spread emerged between November and December, with signs of high viral activity spilling over from the central urban areas to the surrounding areas.

In January and February 2025, the load in the central urban area gradually declined. From April onwards, viral activity resumed in the central urban area and spread outwards, reaching a city-wide epidemic peak in May and June. The high level persisted into July and August. The viral load in the central urban area has been gradually declining since September, but remains at a certain level of prevalence. It should be noted that ever since the peak of Wanzhou District’s viral load reached during May 2025, the district had been maintaining a medium to high level of virus activity (See Figure 3).

### 3.3 Nonlinear associations of DTR, seasonality with wastewater viral load

The wastewater viral load exhibited clear seasonality, characterized by a predominant primary peak alongside two secondary peaks throughout the year. As shown in Figure 3, the seasonally adjusted effect was negative in January, meaning a low activity level. It was steadily rising beginning in February up to a cross-over to the positive effect range, reaching the first minor peak in March, after which there was a slight downward trend in April. Then, it experienced a marked increase and reached the primary peak in June. Following the primary peak, there was a steep fall in the viral load that became negative again in July. The second peak occurred in August and was comparable to the first peak in March, but afterward, the effect was steadily declining to reach its minimum value in October. Finally, from November to December, it had an insignificant rise, but still, remained negative and below the baseline value without a winter peak.

**Figure 4:**
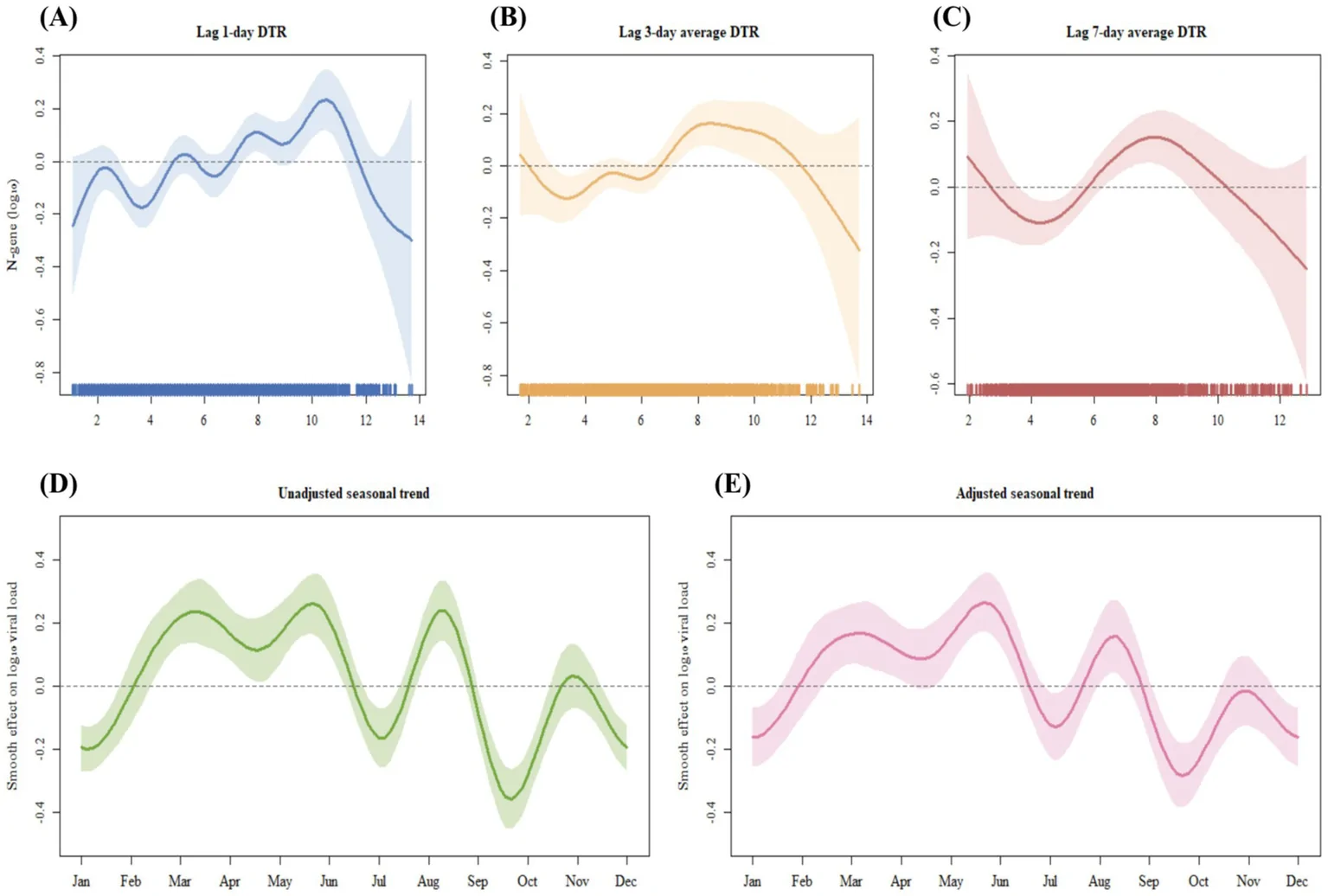
Associations between DTR and wastewater SARS-CoV-2 N gene viral load, and seasonal trends before and after adjustment for DTR. (Top row) Smooth dose–response curves of viral load against lagged DTR estimated by GAMs. The three panels show the effect of **(A)** 1-day lag DTR, **(B)** 3-day lag MA DTR, and **(C)** 7-day lag MA DTR, respectively. The solid line represents the fitted smooth curve, and the shaded area indicates the 95% confidence interval. The horizontal dashed line at zero represents the baseline effect. The rug plots at the bottom show the distribution of observed DTR values. (Bottom row) Seasonal trends of viral load. **(D)** The unadjusted seasonal trend estimated from the base GAM. **(E)** The adjusted seasonal trend after controlling for the nonlinear effect of 7-day lag MA DTR. Both panels show the smooth seasonal effect and its 95% confidence interval over the calendar year.

Both 3-day and 7-day moving average DTR exhibited complex nonlinear relationships with viral loads. While the 1-day model yielded slightly better within-sample fit, its substantially higher effective degrees of freedom (EDF) could raise the possibility of overfitting to sample-specific random noise. When considering both model diagnostic metrics and epidemiological plausibility, the 7-day metric is considered the most robust and statistically stable exposure window (Supplementary Table S6). Wastewater viral load responded positively to DTR within the 6–10 °C window, peaking around 7–8 °C. Outside this range, the relationship reversed, with suppressive effects intensifying as DTR deviated further from the optimum (see Figure 4).

In the fully adjusted GAM, the 7-day lag MA DTR showed a significant nonlinear association with SARS-CoV-2 viral loads in wastewater (EDF = 5.095, *F* = 5.716, *p* < 0.001). Monthly seasonality (EDF = 7.014, *F* = 10.987, *p* < 0.001) and administrative region (EDF = 11.831, *F* = 18.421, *p* < 0.001) also demonstrated significant independent effects. Among wastewater quality parameters, ammonium nitrogen concentration was positively associated with viral loads (*β* = 0.042, SE = 0.016, *p* = 0.008). Compared with 2023, viral loads were significantly lower in 2024 (β = −0.377, SE = 0.075, *p* < 0.001) and 2025 (*β* = −0.226, SE = 0.081, *p* = 0.005). Method comparison across all four viral concentration protocols (PEG precipitation as reference) revealed that viral loads measured by the aluminum flocculation precipitation were significantly lower than those obtained by PEG precipitation (*β* = −0.309, SE = 0.068, *p* < 0.001). By contrast, no statistically significant differences in quantified wastewater SARS-CoV-2 loads were detected for samples processed via centrifugal ultrafiltration (*β* = −0.061, SE = 0.052, *p* = 0.237) or magnetic bead extraction (β = 0.093, SE = 0.059, *p* = 0.115) relative to PEG precipitation. No significant associations were observed for wastewater daily flow, water temperature, total suspended solids, chemical oxygen demand, pH, or resident population coverage (see Table 2).

**Table 2:** The results of the 7-day lag MA DTR-GAM model.

Variable		EDF	*β*	Standard error	t/F	*P*
7-day average DTR		5.095			5.716	<0.001
Month		7.014			10.987	<0.001
Administrative district		11.831			18.421	<0.001
Sampling year	2023		ref			
	2024		−0.377	0.075	−5.005	<0.001
	2025		−0.226	0.081	−2.796	0.005
DWTV			0.128	0.083	1.544	0.123
IWT			−0.036	0.023	−1.544	0.123
SS			0.013	0.025	0.529	0.597
CODCr			0.001	0.015	0.064	0.949
pH			0.007	0.016	0.459	0.646
NH_3_-N			0.042	0.016	2.648	0.008
Inhabitants served			−0.123	0.083	−1.475	0.141
Concentration methods (*n*)	PEG precipitation (1057)		ref			
	Centrifugal ultrafiltration (202)		−0.061	0.052	−1.182	0.237
	Aluminum flocculation precipitation (1100)		−0.309	0.068	−4.560	<0.001
	Magnetic bead method (161)		0.093	0.059	1.576	0.115

* Continuous covariates were standardized by *Z*-score transformation.

## 4 Discussion

Leveraging a long-term wastewater surveillance dataset from Chongqing, this study provides the first empirical evidence of an independent, non-linear association between DTR and community-level SARS-CoV-2 viral loads during the post-pandemic era. To ensure the robustness of this relationship, our modeling approach rigorously adjusted for a comprehensive array of spatial, temporal, methodological, and physicochemical confounders. Furthermore, we characterized a region-specific tri-modal seasonal pattern of viral circulation and quantified the pronounced spatial heterogeneity of viral distribution across the municipality. Collectively, these findings not only reaffirm the efficacy of WBE as a highly sensitive, population-level early-warning tool for SARS-CoV-2 and possibly other respiratory pathogens, but also provide crucial empirical support for integrating meteorological predictors into precision public health interventions.

Based on city-level wastewater surveillance data from Chongqing, SARS-CoV-2 showed three distinct periods of elevated viral signal. One prominent period occurred in late spring (late May–early June), whereas two other periods with comparatively higher viral loads were observed in late winter (late February–early March) and late summer (August). This local profile is partly concordant with external evidence reporting peaks in late summer and winter (40, 41), but differs from studies showing that reported clinical cases remain highest from late autumn through spring (29). Notably, this pattern contrasts with the viewpoint of Ewen Callaway, who observed that ‘3 years after the start of the pandemic, SARS-CoV-2 shows no signs of settling into a seasonal pattern of spread, like influenza has,’ and will instead transition to frequent, variant-driven mini-waves rather than predictable seasonal surges (24). This periodic epidemic pattern is strongly associated with viral evolution and the cyclical fluctuation of variant diversity, rather than single environmental factors. This may be linked to a combination of factors, including temperature and humidity conditions, diurnal temperature fluctuations, indoor gatherings in air-conditioned environments, seasonal rhythms and population mobility (42). Furthermore, the waning of herd immunity and the emergence of variant strains may also influence the intensity and timing of peaks (43–45). These findings suggest that seasonal transmission dynamics may be context-dependent and post-pandemic risk assessment should be locally calibrated using long-term wastewater surveillance data.

Temporal variations in wastewater SARS-CoV-2 loads were generally consistent with officially notified COVID-19 trends across Chongqing. Community transmission remained subdued from September 2024 to April 2025, before a notable resurgence emerged in May 2025 and peaked in June. Wastewater viral levels started to rise as early as March 2025, rising substantially earlier than the rebound in clinically reported cases. This early signal illustrated the capacity of wastewater surveillance to detect a turning point in community transmission ahead of clinical case-based indicators. In the later stages, the reduced virulence of Omicron sublineages and the increase in immune-evading mutations led to a significant rise in the proportion of mild and asymptomatic infections (46). Furthermore, the transition from universal nucleic acid testing to a monitoring model based on voluntary individual testing, coupled with the widespread adoption of home antigen self-testing and the low willingness of those with mild symptoms to seek medical attention and undergo testing, has reduced the proportion of infections captured by official case reports (47). This is consistent with the findings of studies in Europe, and the United States (4, 48–51). Studies conducted in India also corroborate that these factors collectively regulate the strength of the correlation between wastewater and clinical cases (52). In this context, wastewater surveillance offered a complementary source of population-level information that was independent of individual testing and healthcare-seeking behavior (47, 53, 54). As the national positive rate of COVID-19 infections surged and more individuals became willing to undergo treatment during the NB.1.8.1 epidemic in 2025 (55), the reporting of cases became more accurate and the relationship became positive again. These findings illustrated that wastewater surveillance can provide reliable and continuous evidence of community viral circulation in the post-pandemic period, serving as a useful complement to routine case reporting rather than a replacement for it.

Wastewater SARS-CoV-2 viral load serves as a reliable population-level proxy for reflecting real-time community infection burden and trends (20, 21, 56). However, raw wastewater viral signals are susceptible to multi-source confounding biases that hinder accurate assessment of meteorological-driven epidemic fluctuations (57–59). Therefore, this study employed a multi-adjusted GAM framework to partition all potential confounding variations, aiming to clarify the independent relationship between DTR and wastewater SARS-CoV-2 viral loads.

Spatio-temporal heterogeneity, as captured by district-level random intercepts, accounted for the dominant source of variation in wastewater viral loads across Chongqing (see Supplementary Table S7). Clear disparities in viral concentrations were observed among Chongqing’s administrative districts, a geographic pattern repeatedly reported in multi-site wastewater surveillance studies worldwide (47, 59). This spatial heterogeneity likely arises from multiple intersecting determinants, including catchment population composition, socioeconomic characteristics, and viral evolutionary dynamics. Riou et al. demonstrated that age structure correlates with baseline viral signals, with catchments encompassing higher proportions of older population and migrant populations exhibiting systematically higher wastewater viral loads (60). The spatial distribution of circulating variants is another potential contributor. For instance, wastewater surveillance in the Miami area revealed that different dominant variants were present dominant variants differed across geographic sites, accompanied by dynamic competition among variants (61). Given that different SARS-CoV-2 lineages exhibit inherent differences in shedding efficiency (58), such spatial separation of circulating variants would logically translate into differential contributions to total wastewater viral loads across districts.

Beyond demographic and virological spatial confounders, viral concentration protocols constituted another important methodological source of systematic bias in wastewater SARS-CoV-2 quantification, as evidenced by the significant fixed effect of concentration method in our adjusted GAM model. Findings from existing studies consistently show that different pretreatment methods produce significantly different detectable SARS-CoV-2 RNA concentrations even when applied to identical sewage samples (62, 63). Such apparent contradictions can be reconciled by matrix interference effects unique to local sewage. Wastewater physicochemical properties including turbidity, organic matter, and metal ion content alter viral adsorption and precipitation efficiency among protocols, leading to method-specific recovery biases. Although this inherent bias may be difficult to eliminate (60), a nationwide interlaboratory assessment covering 32 U.S. laboratories demonstrated robust reproducibility of SARS-CoV-2 genetic quantification under unified standard operating procedures (64). Minor deviations induced by primer sets and the adoption of RT-qPCR or RT-dPCR platforms contributed far less overall variability than disparities stemming from different viral concentration workflows. In the present study, these method-dependent biases complicate direct cross-district comparisons of absolute viral-load values. Accordingly, interpretations derived from Figure 3 should focus primarily on within-district temporal shifts and spatial–temporal variations of community infection burden, rather than ranking individual districts based on absolute viral-load values.

We incorporated inhabitants served, DWTV, IWT, CODCr, SS, pH, and NH_3_-N into the model to disentangle matrix interference on viral load signals. Among all measured water quality indicators, only NH_3_-N exhibited a statistically significant positive association with wastewater SARS-CoV-2 concentrations. This positive ammonia–virus relationship was also validated by Amoah et al., who additionally found highest SARS-CoV-2 RNA levels at pH 7.1–7.4 (65). Their study also reported inconsistent correlations between total solids and viral signals across different wastewater treatment plants. Such divergence may partially stem from differences in analytical approaches: the earlier study adopted adaptive neuro-fuzzy inference models, while we applied GAM with REML estimation. In line with our observations, an independent wastewater surveillance study similarly reported the absence of a measurable association between influent flow and SARS-CoV-2 loads (66). These outcomes highlight wastewater physicochemical parameters as a dominant water matrix confounder that requires adjustment when exploring environmental correlates of community viral shedding signals.

After adjusting for spatial, temporal, sewage physicochemical, and laboratory methodological confounders, our study identified a significant nonlinear association between 7-day lagged DTR and wastewater SARS-CoV-2 viral loads. This segmented nonlinear pattern shares consistent core observations with previous laboratory and population epidemiological research, which reported the highest viral activity and transmission risk under moderate temperature swings near 7 °C (30), and aligns with regional and global epidemiological evidence documenting elevated COVID-19 transmission under mild diurnal temperature variation (32, 67). Such models focus on the relationship between temperature variation and epidemic incidence or native viruses, while ignoring complex sewage matrices (68, 69), intermittent human viral shedding (70), and time-lag effects (71, 72). Changes in lifestyles such as indoor crowding behaviors and altered ventilation habits that co-occur with DTR are also important potential factors explaining this observed nonlinear association (73, 74). These interpretations remain hypothesis-generating, and causal inference is limited by the ecological design and potential residual confounding.

This study provides guiding procedures for integrating multivariate models into wastewater surveillance. The GAM-based multi-confounder correction enables horizontal and vertical comparability of viral load data across districts, seasons, and laboratory batches by eliminating biases from demographic structure, variant circulation, enrichment methods, and ammonia nitrogen interference. The nonlinear DTR relationship supports meteorology-stratified early warning strategies to help understand and forecast viral fluctuations. Furthermore, spatial heterogeneity findings facilitate differentiated regional risk thresholds and precise public health resource allocation.

There are several drawbacks to this research. Firstly, this study only performed quantitative testing of viral load in wastewater samples and did not involve genomic sequencing of the virus, which means that the study was unable to determine the types of viruses that were circulating in the area. This made it difficult to correlate spatiotemporal distributions with virus variants. Secondly, the open-ended design of the wastewater monitoring cohort limits the conduct of rigorous spatial hotspot analysis, allowing only for intuitive assessments of spatial trends through heatmaps. Finally, PMMoV testing was not initiated until March 2025, preventing retrospective population infection burden estimation for the early phase of our surveillance period.

## 5 Conclusion

In this study, we analyzed temporal and spatial patterns of SARS-CoV-2 RNA in wastewater across Chongqing’s districts and counties to characterize post-pandemic transmission dynamics in a subtropical setting. Our multivariate analysis confirmed that multiple covariates such as administrative districts, viral concentration protocols, wastewater physicochemical indicators, and meteorological factors exerted independent explanatory effects and collectively enhanced the reliability of wastewater viral load modeling. We further identified a significant nonlinear lagged association between DTR and wastewater viral loads. Moreover, spatial heterogeneity of wastewater signals provided empirical evidence to inform differentiated district-level prevention and control strategies. Future multi-site collaborative studies across diverse climatic zones are warranted to verify the generalizability of the DTR effect and refine region-specific meteorological early warning thresholds, ultimately strengthening the evidence base for wastewater-based precision epidemic prevention and control.

## Data Availability

The data analyzed in this study is subject to the following licenses/restrictions: data cannot be made publicly available; readers should contact the corresponding author for details. Requests to access these datasets should be directed to cdczhanghuadong@163.com.
