# Supplementary material for: Wastewater-based epidemiology reveals spatiotemporal dynamics and diurnal temperature range effects on post-pandemic SARS-CoV-2 infection burden in Chongqing, China
Source: Front Public Health. 2026 Aug 26;14:1891965. doi: 10.3389/fpubh.2026.1891965 (PMC13553959; doi:10.3389/fpubh.2026.1891965)
Supplement: Supplementary file 1 [file Supplementary_file_1.docx]

# 1 Supplementary Material

## Text S1. Detailed Laboratory Analytical Protocols for SARS-CoV-2 Quantification in Wastewater

All wastewater detection procedures strictly complied with Chinese national health standards WS/T 799-2022 Method for enrichment and nucleic acid detection of SARS-CoV-2 in sewage and WS/T 10042-2025 Technical specification for surveillance of SARS-CoV-2 in wastewater. The WS/T 799-2022 standard was adopted for wastewater viral nucleic acid detection in the early phase of this study, and all subsequent experimental operations fully followed the updated WS/T 10042-2025 standard after its implementation. Full standardized laboratory workflows, including sample collection, inactivation, method-specific pre-centrifugation, four optional enrichment strategies, RNA extraction, RT-qPCR quantification, and PMMoV recovery correction, are documented in detail below to ensure full methodological reproducibility. Supplementary Table S4 summarizes the PCR instruments, enrichment kits and SARS-CoV-2 detection kits used in each monitoring region.

### S1.1 Wastewater Sample Collection, Transportation and Pre-inactivation

Twenty-four-hour composite wastewater samples were collected from the inlet of wastewater treatment plants or drainage outlets of residential catchments using automatic samplers. Equal-volume subsamples were collected at hourly intervals throughout a full 24 h period to reflect daily averaged viral shedding levels within each service area. All samples were transferred into sterile polyethylene bottles, disinfected externally with 75% ethanol, tightly sealed, and transported to the corresponding local laboratory under continuous refrigeration (0–4 °C). All laboratory pretreatment and enrichment procedures were initiated within 24 h after sampling to avoid viral RNA degradation.

Field blank controls (nuclease-free water processed synchronously alongside field samples) were prepared for each sampling batch to monitor cross-contamination during sampling, transport and pre-processing. Prior to viral enrichment, all wastewater samples and matched blank controls were fully inactivated via incubation in a 60 °C water bath for 30 min, with the water bath liquid level maintained above the surface of sample containers to guarantee complete viral inactivation.

### S1.2 Four Standardized Viral Enrichment Methods

Four viral enrichment approaches specified in Appendix A of WS/T 10042-2025 were utilized. Each laboratory selected one single enrichment method according to its own instrumental configuration and routine testing capacity, rather than parallel testing of all four methods on individual wastewater samples. Pre-centrifugation parameters and judgment criteria differed slightly among the four enrichment workflows, as described separately below for each protocol.

#### S1.2.1 PEG precipitation method

##### Pre-centrifugation pretreatment

For samples with suspended solid mass concentration >400 mg/L, pre-centrifugation was performed at 2000 g for 2 min at 4 °C to remove large particulate debris; the clarified supernatant was retained for subsequent enrichment. Samples with suspended solids ≤400 mg/L skipped this step. Pre-centrifugation may cause minor viral loss, as noted in WS/T 10042-2025.

##### Enrichment procedure

A total of 40 mL pre-clarified wastewater supernatant (raw wastewater if pre-centrifugation was omitted) was mixed with 4.0 g PEG and 0.8 g NaCl, followed by shaking at 150 rpm for 120 min at 4 °C. The mixture was centrifuged at 4800 g for 45 min at 4 °C, and the resulting viral pellet was resuspended in 1 mL nuclease-free water. A secondary centrifugation step (20,000 g, 8 min, 4 °C) was conducted, and approximately 500 μL of concentrated viral suspension was retained for downstream RNA extraction.

#### S1.2.2 Aluminum salt coagulation precipitation method

##### Pre-centrifugation pretreatment

When obvious large visible particles existed in homogenized wastewater or suspended solid mass concentration exceeded 400 mg/L, pre-centrifugation was carried out at 2000 g for 2 min at 4 °C, and the supernatant was collected for coagulation treatment. Samples with low turbidity and low suspended solids were directly used without pre-centrifugation.

##### Enrichment procedure

50 mL wastewater supernatant (untreated raw wastewater for low-solids samples) was transferred into a sterile conical flask, mixed with 0.5 mL 0.3 mol/L AlCl₃ solution, and the pH was adjusted to 6.0 ± 0.1 using 1 mol/L HCl or NaOH solutions. The mixture was shaken at 150 rpm for 15 min at room temperature, then centrifuged at 1900 g for 5 min at 4 °C. After complete removal of supernatant, 0.10 g EDTA disodium salt dihydrate was added to dissolve the virus-laden aluminum hydroxide colloids, followed by incubation in a 60 °C water bath for 10 min.

#### S1.2.3 Centrifugal ultrafiltration method

##### Pre-centrifugation pretreatment

Pre-centrifugation (2000 g, 2 min, 4 °C) was mandatory for wastewater with high suspended solids to prevent rapid membrane blockage during filtration. Low-turbidity samples without obvious particulate impurities could omit pre-centrifugation.

##### Enrichment procedure

Polyethersulfone (PES) ultrafiltration membranes with a molecular weight cutoff of 100 kDa were pre-activated overnight in 75% ethanol before use. A total of 100 mL pre-centrifuged wastewater supernatant (or unprocessed raw wastewater for low suspended solid samples) was filtered under a constant pressure of 0.2 MPa. After filtration was completed, viral particles trapped within the membrane fouling layer were scraped and eluted into 0.8 mL Tris-EDTA buffer (pH = 8.0), generating roughly 1 mL viral concentrate.

#### S1.2.4 Magnetic bead-based nucleic acid enrichment method

##### Pre-centrifugation pretreatment

All samples underwent unified pre-centrifugation at 2000 g for 2 min at 4 °C regardless of suspended solid concentration, to eliminate coarse particles that may interfere with magnetic bead binding and subsequent RNA purification.

##### Enrichment procedure

8 mL pre-centrifuged wastewater supernatant was mixed with 2 mL lysis buffer and 100 μL proteinase K enhancer, then incubated at 56 °C for 15 min to fully lyse viral particles. Hydroxyl-modified magnetic beads were added to specifically bind released viral RNA. After magnetic separation, two sequential rounds of ethanol washing were performed to remove matrix inhibitors, and purified RNA was eluted using 80–200 μL elution buffer.

### S1.3 Viral Nucleic Acid Extraction

RNA extraction was performed on all viral concentrates generated from the four enrichment workflows using commercial RNA extraction kits optimized for complex environmental wastewater matrices, inside Class II biosafety cabinets. Nuclease-free water was included as extraction negative control within every extraction batch to identify contamination during nucleic acid purification. Purified RNA was immediately loaded onto RT-qPCR plates for quantification, or preserved at −80 °C for delayed detection if required. Detailed kit information for each monitoring region is listed in Supplementary Table S4.

### S1.4 RT-qPCR Quantification of SARS-CoV-2 and PMMoV as Internal Standard for Recovery Calculation

#### S1.4.1 Dual-target RT-qPCR for SARS-CoV-2

A dual-target strategy targeting the open reading frame 1ab (ORF1ab) and nucleocapsid (N) genes of SARS-CoV-2 was adopted for all samples.

**Standard curve construction**

Certified SARS-CoV-2 genomic RNA reference material was used to generate calibration curves. Stock standard material was serially diluted to prepare at least five concentration gradients, with the lowest concentration approaching the limit of detection of the detection kit to cover the full concentration range of all wastewater samples. Each concentration gradient was tested in three technical replicates. Standard curves were plotted with cycle threshold (Ct) values on the y-axis and log₁₀-transformed viral copy concentrations on the x-axis, from which amplification efficiency (E) and coefficient of determination (R²) were derived.

**RT-qPCR assay procedure**

Extracted nucleic acid samples were subjected to amplification system preparation and RT‑qPCR detection following the manufacturer’s instructions of the RT‑qPCR kit. Both ORF1ab and N gene targets were detected simultaneously for each sample, with three technical replicates set for each target to reduce random analytical error.

**Quality control and result interpretation**

Standard curves were considered valid only when the amplification efficiency ranged from 90% to 110% and the coefficient of determination (R²) was no less than 0.99; invalid standard curves required re-preparation and re-testing of the corresponding assay plates.

A wastewater sample was defined as SARS-CoV-2 positive if both target genes showed typical S-shaped amplification curves with Ct values within the positive cutoff specified by the kit. For samples with only one target detected, a positive result was confirmed when positive signals appeared in at least two technical replicates. Samples with only one positive replicate for a single target were subjected to re-enrichment, re-extraction and repeated RT-qPCR testing to rule out false-positive signals.

#### S1.4.2 PMMoV quantification for internal standard and recovery calculation

Pepper mild mottle virus (PMMoV) monitoring was initiated in March 2025. As the mandatory endogenous internal standard specified in WS/T 10042-2025, PMMoV was tested synchronously with SARS-CoV-2 for each sample batch. According to the standard Appendix A.3, PMMoV was quantified in both raw untreated wastewater and post-enrichment concentrates, and the PMMoV nucleic acid recovery rate was calculated to evaluate the overall viral loss during the concentration and nucleic acid extraction procedures of individual samples. Identical PMMoV primer and probe sequences outlined in WS/T 10042-2025 were uniformly adopted across all 16 participating laboratories.

PMMoV concentrations were measured in both raw wastewater aliquots and post-enrichment concentrates for each sample. The PMMoV recovery rate (*H*, %) was calculated as:

$$H=\frac{C_{1}}{C_{0}}\times100\%$$

where $C_{0}$ = PMMoV concentration quantified in raw wastewater; $C_{1}$ = PMMoV concentration back-calculated from enriched concentrates. Per the quality control criteria in WS/T 10042-2025, any experimental batch with *H* < 5% was classified as invalid and excluded from subsequent statistical analysis.

#### S1.4.3 Calculation of SARS-CoV-2 viral load in wastewater

The final SARS-CoV-2 nucleic acid concentration in wastewater (copies/mL) was calculated via the unified formula Equation (B.1) provided in Appendix B of WS/T 10042-2025. The PMMoV nucleic acid recovery rate H of each sample was incorporated into this formula to calculate the viral concentration in wastewater, as required by the standard. The full formula is as follows:

$$C=\frac{C_{2}\times V_{4}\times V_{2}}{V_{3}\times V_{1}\times H}$$

where *C* refers to SARS-CoV-2 nucleic acid concentration in wastewater, copies/mL; *C_2_* refers to SARS-CoV-2 concentration in eluted RNA quantified from the RT-qPCR standard curve, copies/mL; *V_4_* refers to elution volume of purified RNA after nucleic acid extraction, mL; *V_2_* refers to volume of viral concentrate obtained after enrichment, mL; *V_3_* refers to volume of viral concentrate used for RNA extraction, mL; *V_1_* refers to volume of wastewater supernatant subjected to viral enrichment, mL; *H* refers to PMMoV nucleic acid recovery rate, %.

All volume parameters were recorded separately for each specimen, and each sample’s own measured recovery rate *H* was applied individually in the calculation, following the standard calculation specification.

Table S1. Expansion of wastewater surveillance coverage and sampling characteristics in Chongqing (2023–2025)

| Time point | Cumulative monitored WWTPs | Service population (10^4^ Inhabitants) | No. of Districts Covered | Districts included |
| --- | --- | --- | --- | --- |
| 2023.2 | 5 | 465.87 | 4 | Nan'an, Beibei, Yubei, Banan |
| 2024.1 | 11 | 791.47 | 9 | Nan'an, Beibei, Yubei, Banan, Dadukou, Jiangbei, Shapingba, Yongchuan, Liangjiangxin |
| 2024.6 | 13 | 938.47 | 11 | Nan'an, Beibei, Yubei, Banan, Dadukou, Jiangbei, Shapingba, Yongchuan, Liangjiangxin, Jiulongpo, Yuzhong |
| 2024.9 | 15 | 1003.47 | 12 | Nan'an, Beibei, Yubei, Banan, Dadukou, Jiangbei, Shapingba, Yongchuan, Liangjiangxin, Jiulongpo, Yuzhong, Wanzhou |
| 2024.10 | 16 | 1052.47 | 13 | Nan'an, Beibei, Yubei, Banan, Dadukou, Jiangbei, Shapingba, Yongchuan, Liangjiangxin, Jiulongpo, Yuzhong, Wanzhou, Hechuan |
| ª2024.11 | 18 | 1122.97 | 15 | Nan'an, Beibei, Yubei, Banan, Dadukou, Jiangbei, Shapingba, Yongchuan, Liangjiangxin, Jiulongpo, Yuzhong, Wanzhou, Hechuan, Jiangjin, Changshou |
| 2025.1 | 19 | 1137.97 | 16 | Nan'an, Beibei, Yubei, Banan, Dadukou, Jiangbei, Shapingba, Yongchuan, Liangjiangxin, Jiulongpo, Yuzhong, Wanzhou, Hechuan, Jiangjin, Changshou, Qianjiang |

ª Sampling was performed twice weekly before this time point, and adjusted to once weekly thereafter.

**Table S2.** Characteristics of Wastewater Treatment Plants.

| **WWTP ID** | **Inhabitants served (10⁴ persons)** | **Sampling frequency** | **DWTV (10^4^ tons/day)** | **pH** | **CODCr（mg/L）** | **NH_3_-N (mg/L)** | **IWT (°C)** | **SS (mg/L)** | **District** |
| --- | --- | --- | --- | --- | --- | --- | --- | --- | --- |
| A | 36 | 232 | 3.47(1.13) | 7.60(0.13) | 213.00(92.25) | 23.40(13.05) | 17.25(5.82) | 113.50(61.25) | Beibei |
| B | 20 | 128 | 3.63(0.75) | 7.60(0.10) | 277.50(118.25) | 32.10(7.65) | 12.50(1.57) | 172.50(51.00) | Beibei |
| C | 66 | 143 | 11.09(1.97) | 7.40(0.20) | 286.00(101.50) | 26.50(9.05) | 21.60(6.60) | 146.00(54.00) | Shapingba |
| D | 50 | 223 | 6.88(1.21) | 7.60(0.10) | 170.00(65.50) | 21.40(5.75) | 22.30(5.15) | 156.00(42.00) | Nan'an |
| E | 20 | 73 | 4.00(0.82) | 7.50(0.20) | 230.00(62.00) | 23.40(7.90) | 21.90(9.70) | 204.00(58.00) | Wanzhou |
| Fª | 129 | 97 | 65.23(8.57) | - | - | - | - | - | Yuzhong |
| G | 31.1 | 143 | 7.15(1.49) | 7.20(0.30) | 198.00(79.50) | 24.10(7.05) | 23.00(8.60) | 102.00(47.00) | Dadukou |
| H | 45 | 73 | 11.00(2.91) | 7.50(0.10) | 207.00(79.00) | 21.40(10.00) | 22.00(9.00) | 185.00(54.00) | Wanzhou |
| I | 41 | 61 | 11.14(1.51) | 7.70(0.10) | 261.00(72.00) | 23.50(8.30) | 19.40(10.00) | 128.00(42.00) | Changshou |
| J | 49 | 63 | 8.11(1.18) | 7.50(0.20) | 210.00(66.00) | 33.10(8.15) | 22.40(8.35) | 108.00(16.00) | Hechuan |
| K | 36.5 | 143 | 6.89(1.44) | 7.60(0.10) | 221.00(105.50) | 24.40(7.55) | 14.20(6.80) | 156.00(123.50) | Liangjiangxin |
| L | 49 | 234 | 6.81(2.52) | 7.80(0.20) | 189.50(75.75) | 23.50(6.20) | 14.25(5.40) | 104.00(55.50) | Yubei |
| M | 60 | 141 | 12.00(0.00) | 7.60(0.40) | 144.00(90.00) | 26.49(10.92) | 17.00(5.00) | 60.00(33.00) | Yongchuan |
| N | 300 | 227 | 103.98(14.87) | 7.60(0.20) | 220.00(117.50) | 22.70(4.45) | 22.70(7.30) | 196.00(145.00) | Nan'an |
| O | 30.87 | 234 | 7.16(1.66) | 7.80(0.20) | 244.00(165.00) | 30.10(9.77) | 16.40(5.85) | 152.50(117.25) | Ba'nan |
| P | 15 | 10 | 2.67(0.85) | 7.70(0.08) | 267.50(112.75) | 25.85(8.43) | 15.15(3.40) | 184.00(109.50) | Qianjiang |
| Q | 112 | 138 | 39.98(5.99) | 7.60(0.20) | 257.50(184.50) | 23.13(6.48) | 23.60(7.95) | 139.00(90.75) | Jiangbei |
| R | 18 | 96 | 2.12(0.59) | 7.24(0.14) | 95.51(64.62) | 36.26(32.53) | 25.11(6.05) | 1634.42(725.30) | Jiulongpo |
| S | 29.5 | 61 | 4.91(0.67) | 7.30(0.10) | 216.00(98.00) | 25.94(11.60) | 22.70(8.10) | 162.00(92.00) | Jiangjin |

Values are median (interquartile range, IQR) over the sampling period. F is a sewage pumping station with no wastewater physical data. ª This site represents a municipal sewage pumping station without wastewater treatment units, which was included in analysis as the exclusive collection point of this district.

**Table S3.** Distribution of lagged DTR values across study wastewater treatment plants.

| WWTP ID | 1-day lagged moving average | | | 3-day lagged moving average | | | 7-day lagged moving average | | | N gene concentration(copies/mL) | | | | log_10_ normalized N gene concentration |
| --- | --- | --- | --- | --- | --- | --- | --- | --- | --- | --- | --- | --- | --- | --- |
|  | min | max | median (IQR) | min | max | median (IQR) | min | max | median (IQR) | Positivity rate(%) | min | max | median (IQR) | median (IQR) |
| A | 1.10 | 13.10 | 6.05(3.75) | 1.60 | 12.13 | 6.05(2.5) | 2.38 | 11.91 | 5.81(2.06) | 66.81 | 0.003 | 219.90 | 1.60(8.58) | 8.22(1.30) |
| B | 1.10 | 13.10 | 6.05(4.5) | 1.60 | 12.13 | 6.02(2.75) | 2.38 | 11.91 | 5.51(2.66) | 78.91 | 0.003 | 212.22 | 4.00(15.61) | 8.95(1.51) |
| C⃰ | 1.50 | 12.65 | 6.05(4.7) | 1.85 | 11.83 | 6.03(2.66) | 2.51 | 11.64 | 5.65(2.3) | 11.89 | 0.500 | 474.00 | 0.50(0.00) | 7.93(0.10) |
| D⃰ | 1.50 | 12.65 | 6.05(4) | 1.85 | 11.83 | 6.15(2.43) | 2.51 | 11.64 | 5.88(2.07) | 71.30 | 0.001 | 105.27 | 2.18(11.21) | 8.49(1.36) |
| E | 2.10 | 13.70 | 7.15(4.95) | 2.30 | 13.90 | 6.4(4.18) | 2.41 | 12.74 | 6.08(2.81) | 78.08 | 0.500 | 419.55 | 8.82(59.74) | 9.32(1.72) |
| F⃰ | 1.50 | 12.65 | 6.4(4.75) | 1.85 | 11.83 | 6.13(2.73) | 2.51 | 11.64 | 5.66(2.39) | 91.75 | 0.003 | 80.98 | 3.28(14.98) | 9.24(1.40) |
| G⃰ | 1.50 | 12.65 | 6.1(4.65) | 1.85 | 11.83 | 6.03(2.74) | 2.51 | 11.64 | 5.65(2.3) | 74.13 | 0.006 | 166.37 | 1.43(8.41) | 8.52(1.25) |
| H | 1.50 | 12.35 | 6.18(4.56) | 1.93 | 12.15 | 5.95(3.76) | 1.94 | 12.09 | 5.74(2.45) | 75.34 | 0.500 | 385.35 | 7.06(33.87) | 9.33(1.57) |
| I | 1.45 | 10.45 | 4.75(4.45) | 1.42 | 8.85 | 5.12(2.68) | 1.81 | 8.54 | 4.8(1.84) | 44.26 | 0.054 | 476.42 | 0.50(27.84) | 8.21(1.81) |
| J | 1.15 | 11.70 | 5.95(5.02) | 1.78 | 10.22 | 5.98(3.12) | 2.59 | 9.22 | 5.49(2.75) | 46.03 | 0.500 | 22.18 | 0.50(4.99) | 8.02(1.03) |
| K | 1.50 | 13.05 | 6(4.9) | 1.82 | 12.77 | 6.1(2.79) | 2.29 | 12.43 | 5.58(2.34) | 82.52 | 0.200 | 117.38 | 2.31(8.26) | 8.64(1.21) |
| L | 1.50 | 13.05 | 6.18(4.24) | 1.82 | 12.77 | 6.15(2.4) | 2.29 | 12.43 | 5.9(2.03) | 67.95 | 0.210 | 84.27 | 2.49(8.68) | 8.55(1.21) |
| M | 1.15 | 13.60 | 6(4.1) | 1.90 | 11.98 | 5.98(2.65) | 2.16 | 11.36 | 5.62(2.21) | 22.70 | 0.500 | 101.65 | 0.50(0.00) | 8.00(0.00) |
| N⃰ | 1.50 | 12.65 | 6.15(4.05) | 1.85 | 11.83 | 6.18(2.42) | 2.51 | 11.64 | 5.89(2.12) | 71.37 | 0.001 | 233.15 | 2.11(15.06) | 8.86(1.49) |
| O | 1.60 | 12.50 | 6.05(4.16) | 1.78 | 11.63 | 5.99(2.51) | 2.55 | 11.40 | 5.78(2.11) | 70.94 | 0.003 | 216.54 | 2.14(10.70) | 8.71(1.37) |
| P | 1.20 | 9.45 | 5.52(2.8) | 2.58 | 10.38 | 4.75(3.68) | 3.34 | 10.36 | 5.18(1.01) | 0.00 | 0.500 | 0.50 | 0.50(0.00) | 7.95(0.13) |
| Q⃰ | 1.50 | 12.65 | 6.05(4.7) | 1.85 | 11.83 | 6.03(2.66) | 2.51 | 11.64 | 5.65(2.3) | 28.26 | 0.310 | 27.17 | 0.50(0.28) | 8.27(0.20) |
| R⃰ | 1.50 | 12.20 | 6.1(4.61) | 1.85 | 11.83 | 6.03(2.7) | 2.51 | 11.44 | 5.63(2.26) | 89.58 | 0.001 | 94.61 | 5.45(22.24) | 8.75(1.45) |
| S | 1.65 | 11.75 | 5.45(4.85) | 1.90 | 8.68 | 5.67(3.27) | 2.68 | 8.85 | 5.44(2.46) | 9.84 | 0.500 | 61.30 | 0.50(1.46) | 7.94(0.70) |

WWTP ID marked with ⃰ were assigned data from the Shapingba weather station due to their close proximity and overlapping urban catchments.

**Table S4. Laboratory workflows for viral enrichment, RNA extraction and RT-qPCR at district wastewater surveillance sites, Chongqing, China**

| **District** | **Viral enrichment method** | **Extractor brand** | **Extraction kit brand** | **Elution volume (μL)** | **RT-qPCR platform brand** | **SARS-CoV-2 detection kit brand** |
| --- | --- | --- | --- | --- | --- | --- |
| Beibei | Aluminum flocculation precipitation | Zybio | Zybio | 60 | Anyu | MABSKY |
| Shapingba | Aluminum flocculation precipitation | Zybio | Zybio | 60 | Thermo Fisher | EDIAGNOSIS |
| Nan'an | Centrifugal ultrafiltration | Zybio | Zybio | 60 | Bioer | MABSKY |
| Wanzhou | Aluminum flocculation precipitation | BioPerfectus | BioPerfectus | 85 | Bioer | MABSKY |
| Yuzhong | PEG precipitation | BioPerfectus | BioPerfectus | 80 | Bio-Rad | XABT |
| Dadukou | PEG precipitation | BioPerfectus | BioPerfectus | 60 | Bioer | MABSKY |
| Changshou | Aluminum flocculation precipitation | Zybio | Zybio | 50 | Thermo Fisher | EDIAGNOSIS |
| Hechuan | Magnetic bead-based enrichment | BioPerfectus | BioPerfectus | 60 | Bioer | BioPerfectus |
| Liangjiangxin | PEG precipitation | HUIDUDAYUAN | HUIDUDAYUAN | 80 | Thermo Fisher | HUIDUDAYUAN |
| Yubei | PEG precipitation | HUIDUDAYUAN | HUIDUDAYUAN | 80 | Thermo Fisher | HUIDUDAYUAN |
| Yongchuan | Aluminum flocculation precipitation | BioPerfectus | BioPerfectus | 60 | Bio-Rad | MABSKY |
| Ba'nan | Aluminum flocculation precipitation | BioPerfectus | Zybio | 80 | Bio-Rad | XABT |
| Qianjiang | Centrifugal ultrafiltration | BioPerfectus | BioPerfectus | 60 | Bio-Rad | MABSKY |
| Jiangbei | Aluminum flocculation precipitation | BioPerfectus | BioPerfectus | 50 | Tianlong | MABSKY |
| Jiulongpo | PEG precipitation | BioPerfectus | Zybio | 80 | Bio-Rad | XABT |
| Jiangjin | Aluminum flocculation precipitation | BioPerfectus | BioPerfectus | 60 | Bioer | MABSKY |

**Table S5. Manufacturer index for experimental reagents and equipment**

| **Abbreviation** | **Full brand name (Chinese)** | **Full official English brand name** | **Official website** |
| --- | --- | --- | --- |
| Zybio | 中元汇吉 | Zybio Inc. | [https://www.zybio.com](https://link.wtturl.cn/?target=https%3A%2F%2Fwww.zybio.com&scene=im&aid=582478&lang=zh) |
| BioPerfectus | 硕世生物 | BioPerfectus Technologies Co., Ltd. | [https://www.bioperfectus.cn](https://link.wtturl.cn/?target=https%3A%2F%2Fwww.bioperfectus.cn&scene=im&aid=582478&lang=zh) |
| Huidudayuan | 恢度达源 | Huidudayuan Biotechnology Co., Ltd. | [http://huidudayuan.com](https://link.wtturl.cn/?target=http%3A%2F%2Fhuidudayuan.com&scene=im&aid=582478&lang=zh) |
| MABSKY | 生科原 | MABSKY Bio-Tech Co., Ltd. | [http://mabsky.com/](https://link.wtturl.cn/?target=http%3A%2F%2Fmabsky.com%2F&scene=im&aid=582478&lang=zh) |
| EDIAGNOSIS | 明德生物 | Wuhan Easy Diagnosis Biomedicine Co., Ltd. (eDiagnosis) | [http://www.mdeasydiagnosis.com](https://link.wtturl.cn/?target=http%3A%2F%2Fwww.mdeasydiagnosis.com&scene=im&aid=582478&lang=zh) |
| XABT | 卓诚惠生 | Beijing Applied Biological Technologies Co., Ltd. (XABT) | [https://x-abt.com](https://link.wtturl.cn/?target=https%3A%2F%2Fx-abt.com&scene=im&aid=582478&lang=zh) |
| Bioanyu | 安誉 | Bioanyu | [http://www.bioanyu.com/](https://link.wtturl.cn/?target=http%3A%2F%2Fwww.bioanyu.com%2F&scene=im&aid=582478&lang=zh) |
| Bioer | 博日 | Bioer Technology Co., Ltd. | [https://www.bioer.com.cn](https://link.wtturl.cn/?target=https%3A%2F%2Fwww.bioer.com.cn&scene=im&aid=582478&lang=zh) |
| Bio-Rad | 伯乐 | Bio-Rad Laboratories, Inc. | [https://www.bio-rad.com](https://link.wtturl.cn/?target=https%3A%2F%2Fwww.bio-rad.com&scene=im&aid=582478&lang=zh) |
| Thermo Fisher | 赛默飞 | Thermo Fisher Scientific (Applied Biosystems) | [https://www.thermofisher.com](https://link.wtturl.cn/?target=https%3A%2F%2Fwww.thermofisher.com&scene=im&aid=582478&lang=zh) |
| Tianlong | 天隆 | Xi’an Tianlong Science & Technology Co., Ltd. | [https://www.tianlong.com](https://link.wtturl.cn/?target=https%3A%2F%2Fwww.tianlong.com&scene=im&aid=582478&lang=zh) |

| Table S6. Goodness-of-fit comparison of three lagged DTR-GAM models | | | | | |
| --- | --- | --- | --- | --- | --- |
| Lag Window | Deviance explained(%) | Adjusted R^2^ | GCV | EDF | *P*-value |
| 1-day | 21.09 | 0.198 | 2154.811 | 8.03 | ＜0.001 |
| 3-day average | 20.09 | 0.189 | 2159.115 | 4.9 | ＜0.001 |
| 7-day average | 20.19 | 0.19 | 2157.241 | 5.09 | ＜0.001 |

| **Table S7. Deviance explained across stepwise adjusted GAMMs** | | |
| --- | --- | --- |
| Model | Included variables composition | Deviance explained(%) |
| M1 | District random effect | 10.004 |
| M2 | M1 + Concentration Method | 11.205 |
| M3 | M2 + Wastewater physicochemical covariates | 11.631 |
| M4 | M3 + 7-day lag DTR smooth term & Seasonal smooth term & Year | 20.186 |
